# Modeling the Cognitive Development Based on Fine Motor Skills in Preterm and Full‐Term Toddlers Using Lasso Regression

**DOI:** 10.1002/brb3.70485

**Published:** 2025-04-18

**Authors:** Ramazan Yildiz, Ayse Yildiz, Onur Camli, Bulent Elbasan

**Affiliations:** ^1^ Department of Physical Therapy and Rehabilitation, Faculty of Health Sciences Erzurum Technical University Erzurum Turkey; ^2^ Department of Mathematics, Faculty of Science Erzurum Technical University Erzurum Turkey; ^3^ Department of Physical Therapy and Rehabilitation, Faculty of Health Sciences Gazi University Ankara Turkey

**Keywords:** cognitive development, fine motor skills, preterm toddlers

## Abstract

**Background:**

The purpose of this study was to investigate the relationship between fine motor skills and cognitive development in preterm and term toddlers aged 12–15 months.

**Methods:**

A total of 150 participants, 79 preterm, and 71 term toddlers, were assessed using the Bayley Scales of Infant and Toddler Development, Third Edition (Bayley‐III), for cognitive development and the Peabody Developmental Motor Scales, Second Edition (PDMS‐2), for fine motor skills. The relationship between fine motor skills and cognitive development was examined with the lasso regression model.

**Results:**

The study revealed that fine motor skills, particularly grasping, significantly influence cognitive development, with preterm toddlers demonstrating lower scores compared to term peers. Additionally, prenatal and perinatal factors, including gestational age and birth weight, were found to correlate with cognitive outcomes.

**Conclusions:**

These findings emphasize the importance of integrating motor skill‐based interventions into early childhood programs to enhance cognitive and overall developmental outcomes. Future research should explore the causal mechanisms underlying this relationship.

## Graphical abstract

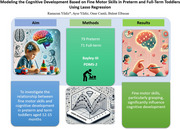

## 1 Introduction

Premature birth is defined as birth before 37 weeks of gestation. Most preterm infants have a higher risk of death than their full‐term counterparts, and those who survive often have physical, cognitive, and emotional problems, with problems in cognition, attention, learning, and behavior (Johnson 2007, Wolke et al. 2008, Salt and Redshaw 2006, Lawn et al. 2013).

The most common problems encountered in premature children who do not develop cerebral palsy (CP) include weakness in gross and fine motor skills (de Kieviet et al. 2009, Williams et al. 2010). Certain types of brain injury, such as global, widespread white matter damage, hemorrhages affecting the motor cortex and corticospinal tract, and ischemic lesions common in preterm infants, can cause disruptions in fine motor networks (Volpe 2009). Preterm children often exhibit impairments in multiple developmental domains, such as executive functions, visual perception, and visuospatial information processing. This complex neurocognitive comorbidity across numerous domains may impair the performance of fine motor tasks in daily life (Potharst et al. 2011).

Fine motor skills are an essential component of activities of daily living and support self‐care activities such as dressing and eating. Moreover, fine motor skills are the basis for skilled play activities and manipulation of toys or objects (Eisert and Lamorey 1996). Poor fine motor skills can lead to increased anxiety, poor academic achievement, and low self‐esteem (Gaul and Issartel 2016). Studies with typically developing children have found that fine motor skills are associated with cognitive abilities (Van der Fels et al. 2015, Martzog et al. 2019). Given that fine motor development is essential for object manipulation and exploring the world, it is expected to contribute to cognitive growth through sensory‐motor experiences and problem‐solving opportunities. In preterm children, delayed development of fine motor skills may impede this exploratory learning and potentially affect cognitive outcomes. However, the extent of this relationship remains to be determined, especially during the early gait period. This study hypothesizes that fine motor skills, particularly grasping ability, significantly influence cognitive development in toddlers, with preterm children exhibiting lower cognitive scores compared to their full‐term peers. We further hypothesize that prenatal and perinatal factors, such as gestational age and birth weight, modulate this relationship. Understanding these associations is crucial for developing targeted early interventions aimed at enhancing both motor and cognitive outcomes in preterm children. By employing a robust statistical approach, the lasso regression model, this study aims to identify key predictors of cognitive development and contribute to evidence‐based early childhood intervention strategies.

## 2 Methods

### 2.1 Participants

This study was conducted on 150 children aged 12–15 months (corrected age). The participants consisted of two groups: 79 preterm (gestational age <37 weeks) and 71 term (gestational age ≥37 weeks). The study inclusion criteria were as follows: Infants with a corrected age of 12–15 months who had not had a neurological diagnosis (e.g., cerebral palsy) or a genetic diagnosis (e.g., Down syndrome) and who had started to walk independently were included in the study. Participants were determined in accordance with these criteria and age and gender were matched between the groups.

Before the study, written consent was obtained from the families and ethics committee approval was obtained (Number of meetings: 11, number of decisions: 7, date: 10.10.2024). This research was performed from October 2024 to January 2025 at Erzurum Technical University, Faculty of Health Sciences. Evaluations were conducted by a physiotherapist possessing 11 years of experience. The cognitive development of the infants was evaluated using the Bayley Scales of Infant and Toddler Development, Third Edition (Bayley‐III), while their fine motor skills were assessed with the Peabody Developmental Motor Scales, Second Edition (Peabody‐II). The assessment environment was arranged quietly and calmly to avoid distracting the infants' attention. The corrected age, gestational age, information about birth, and parental education status of all infants were recorded.

### 2.2 Assessment of Fine Motor Skills

This study utilized the Peabody Developmental Motor Scales, Second Edition (PDMS‐2) to evaluate children's fine motor skills, and the Fine Motor Quotient (FMQ) was computed for these skills. PDMS‐2 is a standardized instrument intended to evaluate the motor development stages of children aged 0–5 (Folio 1983). Fine motor skills were evaluated using two fundamental subtests of the scale:

Grasping: This subtest evaluates the child's capacity to grasp, release, and manipulate objects with their hands. Motor control and hand use developmental levels were assessed through age‐appropriate tasks tailored to the child's developmental stage.

Visual‐motor integration: This subtest assesses the child's capacity to synthesize visual stimuli with motor responses. Tasks encompassed activities such as sketching, constructing with blocks, and arranging objects.

Raw scores from both subtests were standardized in accordance with PDMS‐2 guidelines, and the Fine Motor Quotient (FMQ) was computed (Folio 1983). FMQ is a composite metric that facilitates the comparison of a child's fine motor skills against age‐appropriate norms and serves as the primary analytical variable for assessing fine motor development in this study.

### 2.3 Assessment of Cognitive Development

This study employed the cognitive subscale of the Bayley Scales of Infant and Toddler Development—Third Edition (Bayley‐III) to evaluate the cognitive development levels of children. The Bayley‐III is a highly valid and reliable measurement instrument that evaluates the developmental performance of infants and young children aged 1 to 42 months (Albers and Grieve 2007). The cognitive subscale assesses the child's abilities in cognitive processes including problem‐solving, object permanence, spatial relations, memory, attention, and learning.

The evaluation was conducted individually by a certified professional adhering to a standardized protocol. A tranquil and serene environment was favored to reduce the impact of distracting factors during the measurement. The cognitive subscale comprises age‐appropriate tasks, with each task evaluated based on the child's performance. The raw scores from the scale were standardized based on age norms and transformed into composite scores (Bayley 2009). This study employed cognitive composite scores to assess the cognitive development levels of children.

### 2.4 Statistical Analysis

Descriptive statistics for continuous variables were summarized as mean ± standard deviation (SD), while categorical variables were presented as counts and percentages. To visually explore the relationships between variables, a multiple correlation graph was constructed.

To investigate the difference between two groups (preterm and full‐term), for continuous variables, Student's *t*‐tests (Biometrika 1908) were employed when the assumption of normality was satisfied, as determined by diagnostic methods such as the Shapiro–Wilk test (Shapiro and Wilk 1965). The Wilcoxon Rank‐Sum test (Mann–Whitney *U* test) (Mann and Whitney 1947) was used for continuous variables that did not meet the normality assumption. When categorical variables are concerned, chi‐square tests (Pearson 1900) were conducted to evaluate difference between two groups.

Lasso (Least Absolute Shrinkage and Selection Operator) regression (Tibshirani 1996) was employed to model the relationship between the cognitive variable and a set of predictors. Lasso regression, introduced by Tibshirani, is a type of penalized regression that combines variable selection and regularization to enhance model interpretability and predictive performance (Tibshirani 1996). The Lasso method minimizes the residual sum of squares subject to a constraint on the sum of the absolute values of the regression coefficients. This constraint introduces an L1 penalty, which forces some of the regression coefficients to shrink exactly to zero, effectively performing variable selection.

The mathematical formulation of Lasso regression is as follows:

$$\mathrm{minimize}\sum_{i=1}^{N}{\left(y_{i}-\beta_{0}-\sum_{j=1}^{p}\beta_{j}x_{ij}\right)}^{2}+\lambda \sum_{j=1}^{p}\left|\beta_{j}\right|,$$

where $y_{i}$ is the response variable for the ith observation, $x_{ij}$ represents the jth predictor for the ith observation, $\beta_{0}$ is the intercept, $\beta_{j}$ are the regression coefficients, λ is the tuning parameter that controls the degree of shrinkage.

The value of the penalty parameter λ is selected using cross‐validation, typically through methods like *k*‐fold cross‐validation, to optimize the trade‐off between bias and variance. Lasso regression is particularly beneficial when dealing with high‐dimensional data where multicollinearity among predictors exists or when variable selection is required.

Model performance metric such as the coefficient of determination ($R^{2}$), were used to evaluate the model's fit and predictive ability.

In this study, all data cleaning, preprocessing, and statistical analyses were performed using the R programming language (Team 2020). The correlation graph was produced using the *corrplot* package (Wei et al. 2017). The *glmnet* package was used to fit the Lasso regression model (Friedman et al. 2010). The *cv.glmnet* function was applied to identify the optimal λ value through cross‐validation.

We present the findings of our study, organized into two main parts: Exploratory Data Analysis (EDA) and Modeling. The EDA provides an overview of the data, summarizing the differences between preterm and full‐term groups for both categorical and numerical variables. Statistical tests, including the Pearson's chi‐square test for categorical variables and the Wilcoxon Rank‐Sum (Mann–Whitney U) test for numerical variables, were used to determine the significance of group differences as these variables did not satisfy the normality assumption based on statistical tests (e.g., Shapiro–Wilk test; Shapiro and Wilk 1965). Additionally, a correlation analysis was performed to examine the strength and direction of linear relationships between variables, and the results are visualized in a correlation plot.

Following the EDA, we performed LASSO regression analysis to identify significant predictors and reduce model complexity. Cross‐validation was employed to select the optimal regularization parameter (λ), balancing prediction accuracy and model interpretability. The results include a visual representation of the tuning process and a summary of the final model coefficients.

The findings provide valuable insights into the relationships between various predictors and the cognitive variable, highlighting key factors influencing the results.

## 3 Results

The study was completed with a total of 150 infants (79 preterm and 71 full‐term). There was no difference in age between the preterm and full‐term groups (*p* = 0.184, mean ages were 12.27 ± 0.67 and 12.42 ± 0.64 months, respectively). Gestational ages were 31.43 ± 3.15 weeks in the preterm group and 38.70 ± 1.24 weeks in the full‐term group.

### 3.1 Explanatory Data Analysis

The frequency and percentages of categorical variables for the two groups, along with *p*‐values from the Pearson's chi‐square $(\chi^{2}$) test are presented in Table 1. The comparison of numerical variables between the two groups, with *p*‐values obtained using the Wilcoxon rank sum test (Mann–Whitney test) is presented in Table 2.

**TABLE 1:** Frequency and percentages of two groups based on categorical factors.

Variable	Levels	Preterm (*n* = 79) *n* (%)	Full‐term (*n* = 71) *n* (%)	Total (*n* = 150) *n* (%)	*p* value
Gender	Girl	40 (50.00%)	40 (50.00%)	80 (53.33%)	0.484
	Boy	39 (55.71%)	31 (44.29%)	70 (46.67%)	
Mom education	< 12 years	23 (37.10%)	39 (62.90%)	62 (41.33%)	0.001*
	≥ 12 years	56 (63.64%)	32 (36.36%)	88 (58.67%)	
Father education	< 12 years	15 (27.78%)	39 (72.22%)	54 (36.00%)	<0.001*
	≥ 12 years	64 (66.67%)	32 (33.33%)	96 (64.00%)	
Pregnancy type	Normal	58 (46.40%)	67 (53.60%)	125 (83.33%)	<0.001*
	IVF	21 (84.00%)	4 (16.00%)	25 (16.67%)	
Method of birth	C/S	36 (46.75%)	41 (53.25%)	77 (51.33%)	0.136
	SVD	43 (58.90%)	30 (41.10%)	73 (48.67%)	
Multiple pregnancy	Yes	7 (53.85%)	6 (46.15%)	13 (8.67%)	0.929
	No	72 (52.55%)	65 (47.45%)	137 (91.33%)	

IVF: in vitro fertilization, SVD: spontaneous vaginal delivery, C/S: caesarean section.

∗Significance at the significance level of 0.05 for Pearson's chi‐square ($\chi^{2}$) test.

**TABLE 2:** Comparison of two groups based on numerical factors.

Variable	Preterm (mean ± SD) (*n* = 79)	Full‐term (mean ± SD) (*n* = 71)	*p* value
Mom age (year)	30.58 ± 4.52	28.44 ± 4.67	<0.001*
Father age (year)	32.68 ± 4.18	31.65 ± 4.81	0.139
Birth weight (gram)	1586.00 ± 578.14	3274.00 ± 278.04	<0.001*
Head circumference (cm)	28.24 ± 2.26	36.85 ± 0.82	<0.001*
Gravida (*n*)	1.59 ± 0.68	2.06 ± 0.77	<0.001*
Parite (*n*)	1.49 ± 0.52	1.61 ± 0.54	<0.004*
Abortus (*n*)	0.37 ± 0.37	0.60 ± 0.52	<0.001*
Gestational age (week)	31.43 ± 3.15	38.70 ± 1.24	<0.001*
Cognitive score	104.49 ± 11.89	110.00 ± 15.02	<0.001*
Fine motor score	98.62 ± 11.47	105.80 ± 12.68	<0.001*
Visual motor score	57.10 ± 10.89	65.56 ± 7.82	<0.001*
Grasping score	37.04 ± 5.22	38.99 ± 6.39	<0.001*

SD: standard deviation.

∗Significance at the significance level of 0.05 for Wilcoxon rank sum test (Mann–Whitney) test.

Significant differences exist for mom education, father education, and pregnancy type between preterm and full‐term groups (*p* < 0.05). Gender, method of birth, and multiple pregnancy do not differ significantly between the groups.

Significant differences exist between the two groups for most numerical variables, including maternal age, birth weight, head circumference, gravida, parite, abortus, gestational age, and motor/cognitive skills (*p* < 0.05). Father Age is the only variable without a significant difference. Full‐term group demonstrated significantly higher values for birth weight, head circumference, gestational age, and cognitive/motor skills. Preterm group had significantly older maternal ages and lower gravida scores.

**FIGURE 1:**
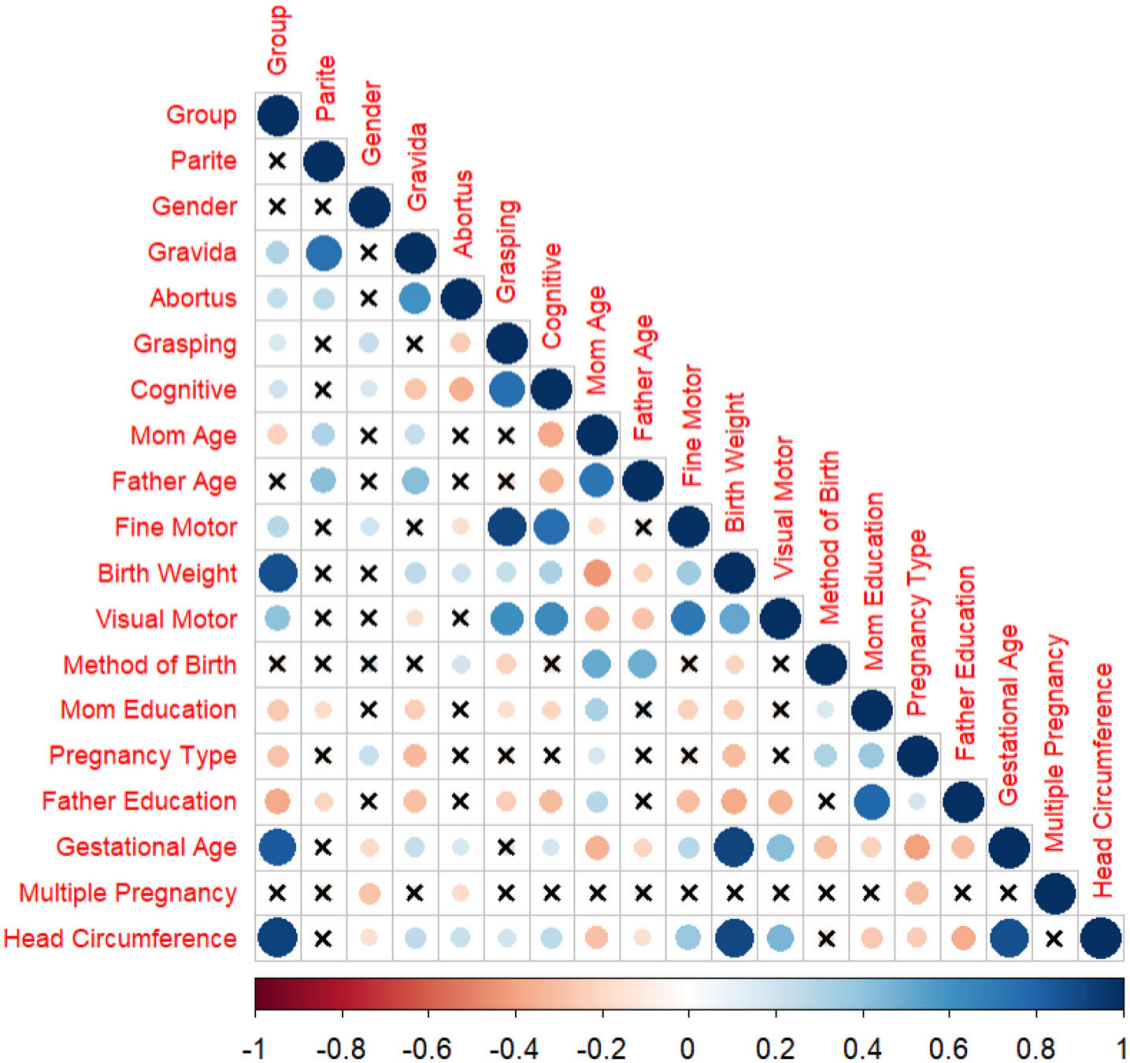
Correlation matrix.

The provided correlation plot in Figure 1 visually represents the pairwise correlations between variables. The correlation coefficients are depicted using color intensity and circle size. In this plot, blue shades indicate positive correlations (closer to +1), while red shades represent negative correlations (closer to –1). White or pale areas indicate weak or no correlation (around 0). The size of each circle corresponds to the strength of the correlation: larger circles indicate stronger relationships, while smaller circles represent weaker correlations. X‐marked cells represent non‐significant correlations, meaning that the observed relationships may not be statistically meaningful.

There is a strong positive correlation between parite and gravida, fine motor and grasping, gestational age and birth weight, head circumference and birth weight, head circumference and gestational age as indicated by a large blue circle. Also, there is moderate positive correlation between abortus and gravida, fine motor and cognitive, visual motor and grasping, visual motor and cognitive, visual motor and fine motor, father education and mom education.

A few red circles (e.g., between abortus and cognitive, mom age and cognitive, mom age and birth weight, gestational age and pregnancy type) indicate weak‐moderate negative correlations. Non‐significant relationships are scattered across the plot, as indicated by the X‐marked cells.

### 3.2 Modelling

**FIGURE 2:**
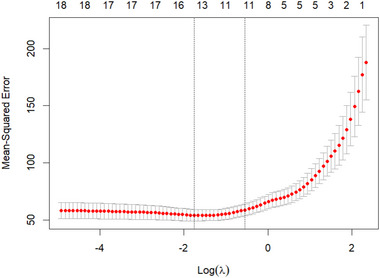
Mean‐squared error vs. log(𝜆).

To identify the optimal value of the regularization parameter 𝜆, cross‐validation was performed. The relationship between Mean Squared Error (MSE) and log(𝜆) is visualized in Figure 2.

The dashed vertical lines represent the 𝜆 values. The left line corresponds to the 𝜆 with the minimum MSE (𝜆 = 0.157). The right line follows the 1‐Standard Error rule, selecting a simpler model with slightly higher error. The final lasso model is obtained with 𝜆 = 0.157.

The Lasso regression retained the following predictors as significant, with their coefficients presented in Table 3.

**TABLE 3:** Lasso regression coefficients.

Variable	Coefficient
Intercept	79.859
Gender	—
Mom education	—
Father education	−0.376
Pregnancy type	1.144
Method of birth	5.434
Multiple pregnancy	−3.579
Group	—
Mom age	−0.826
Father age	−0.235
Birth weight	0.003
Head circumference	—
Gravida	−1.263
Parite	2.854
Abortus	−7.139
Gestational age	−0.275
Fine motor	0.272
Visual motor	—
Grasping	0.964
*R* **^2^**	0.778

Variables such as pregnancy type (1.144), method of birth (5.4341), birth weight (0.003), parite (2.854), fine motor (0.272) and grasping (0.9640) positively influenced the cognitive. Factors like father education (–0.376), multiple pregnancy (–3.579), mom age (–0.826), father age (–0.235), gravida (–1.2637), abortus (–7.1392), and gestational age (–0.275) showed a negative effect on the cognitive. Non‐significant predictors, such as gender, mom education, group, head circumference and visual motor were removed by the Lasso penalty.

The Lasso regression model explains 77.8% of the variability in the cognitive variable, indicating strong model performance. The model effectively balances accuracy and simplicity, reducing the risk of overfitting while retaining key predictors.

## 4 Discussion

This study evaluated the relationship between fine motor skills, prenatal and perinatal factors, and cognitive development in 12‐ to 15‐month‐old preterm and full‐term toddlers. The findings revealed that fine motor skills play an important role in cognitive development and emphasized the close relationship between motor skills and cognitive abilities. In addition, prenatal and perinatal factors were found to significantly affect cognitive skills. These results suggest that motor and cognitive development in early childhood progress in parallel and that environmental and biological factors play an important role in this process.

It has been widely documented in the literature that preterm infants have poorer cognitive and motor development compared to term infants (Khurana et al. 2020). Maggi et al. (2014) found that preterm infants had lower motor coordination, cognitive and functional performance than term infants at 4 years of age. Regarding fine motor skills, evidence from studies using standardized assessments revealed that preterm infants achieved lower eye‐hand coordination scores than their full‐term peers between 1 and 24 months of age and that this difference increased significantly over time (Sansavini et al. 2011, Yaari et al. 2018). In another study, Sansavini et al. (2014) reported that extremely preterm infants had lower cognitive and fine motor scores than full‐term infants at 12, 24, and 30 months. Moderate to severe delays in fine motor development of extremely preterm infants in the first years of life were reported to persist at 30 months of age (Månsson and Stjernqvist 2014). In a comprehensive meta‐analysis, it was emphasized that preterm children had more motor problems and poorer cognitive skills compared to their term peers from preschool to school age (Allotey et al. 2018). Consistent with the literature, both cognitive development and fine motor skills of preterm infants were lower than their full‐term peers in the present study. The fact that preterm infants exhibit poorer motor and cognitive performance compared to term infants indicates the negative effects of preterm birth on neurodevelopmental processes. These findings emphasize the importance of a multidisciplinary approach to detect and intervene early in the developmental delays of preterm infants.

Manual exploration provides learning opportunities that influence cognition in children by manipulating objects (Thelen 2000). In many studies, fine motor skills have been associated with children's cognitive and academic development (Martzog 2015, Suggate and Stoeger 2017). In terms of cognitive skills, FMS has been associated with reasoning, working memory, executive function and intelligence in preschool children (Martzog 2015, Becker et al. 2014, Cameron et al. 2012). Regarding academic development, FMS has been emphasized as an important factor in school readiness (Morrison and Hindman 2012). Although there are studies in the literature that draw attention to this relationship in the preschool period, it has not been examined in toddlers. In this study conducted in preterm and term children in early childhood, fine motor skills, especially grasping, were found to affect cognitive development. This finding is consistent with the existing literature supporting the fundamental role of motor skills in the process of interacting with the environment and structuring cognitive processes (Roebers et al. 2014). This relationship between fine motor skills and cognitive abilities suggests that engaging toddlers in motor skill‐based tasks at an early stage can increase learning opportunities. For example, activities such as playing with blocks or stringing beads can simultaneously improve children's fine motor skills and cognitive functions such as problem solving and categorization. However, it is not entirely clear what drives this relationship; for example, whether dexterity promotes cognitive development or whether children with better cognitive skills are more successful at more complex motor tasks is a question that needs to be addressed in future research.

The impact of prenatal and perinatal factors on children's cognitive development around 1 year of age suggests that neurodevelopmental outcomes are vulnerable to both genetic and environmental interactions. In our study, variables such as paternal education, pregnancy type, delivery method, multiple pregnancy, maternal age, paternal age, birth weight, gravidity, parity, abortion and gestational age were found to be associated with cognitive development. In the literature, it is emphasized that low birth weight and short gestational age may negatively affect neurodevelopmental development (Largo et al. 1989) Factors such as advanced maternal and paternal age have been reported to affect the cognitive potential of children by increasing the risk of complications during the birth process (Tearne 2015, Saha et al. 2009). In addition, socioeconomic factors such as parental education level have been found to play a determinant role in the cognitive development of children and low education level may cause developmental delays (Phillips and Shonkoff 2000). The impact of prenatal and perinatal factors on the cognitive development of preterm children is multifaceted and each of these factors plays a critical role in shaping children's neurodevelopmental outcomes. In this context, early interventions and parent education programs are crucial to support children's healthy development.

The study's limitations encompass its cross‐sectional design, which permitted only the analysis of associations, and the minimal variability among the results. The standards for interpreting gross motor skill scores and cognitive assessments on the PDMS‐2 and Bayley‐III instruments are derived from samples in the United States. Given that these are the sole available norms, certain results should be interpreted cautiously due to variations in cultural context.

## Author Contributions

**Ramazan Yildiz**: conceptualization, investigation, writing–original draft, methodology, data curation. **Ayse Yildiz**: conceptualization, investigation, writing–review and editing, writing–original draft, data curation. **Onur Camli**: formal analysis, visualization, methodology, writing–review and editing, writing–original draft. **Bulent Elbasan**: writing–review and editing, supervision.

## Ethics Statement

Permission was received from the Erzurum Technical University Ethics Committee to conduct the study (Number of meetings: 11, number of decisions: 7, date: 10.10.2024). Parents were asked to sign a consent form to allow their infants to participate in the study.

## Conflicts of Interest

The authors declare no competing interests.

### Peer Review

The peer review history for this article is available at https://publons.com/publon/10.1002/brb3.70485

## Data Availability

The datasets used and/or analyzed during the current study are available from the corresponding author upon reasonable request.
